# Characterization and applications of chimeric mice with humanized livers for preclinical drug development

**DOI:** 10.1186/s42826-019-0032-y

**Published:** 2020-01-08

**Authors:** Chise Tateno, Yuha Kojima

**Affiliations:** 1 Research and Development Department, PhoenixBio Co., Ltd., 3-4-1 Kagamiyama, Higashihiroshima, Hiroshima, 739-0046 Japan; 2 Research Center for Hepatology and Gastroenterology, Hiroshima University, 1-2-3 Kasumi, Minami-ku, Hiroshima, Hiroshima 734-0037 Japan

**Keywords:** Chimeric mice, Human hepatocytes, DMPK, HBV, HCV, Toxicology

## Abstract

We have succeeded in stable mass production of chimeric PXB-mice, whose liver is repopulated by human hepatocytes at a ratio of more than 70%, and we are providing these mice to academia and pharmaceutical companies to support the development of new drugs or studies of liver function. Furthermore, we isolated human hepatocytes, called PXB-cells, from the chimeric mice, and provide them for clients weekly for in vitro studies. In this review, we summarize the existing characterizations of PXB-mice and PXB-cells and their present and future applications.

## Introduction

Albumin enhancer promoter-driven urokinase plasminogen activator transgenic mice (uPA-Tg mice) were produced by Heckel et al. in 1990 [1]. Originally, these mice were produced to investigate the physiological role of uPA in vivo. However, the researchers noticed that the mouse liver was damaged by high expression of uPA and could be repopulated by transplanting healthy mouse hepatocytes via spleen [2]. uPA is a kind of serine protease that is produced in mouse hepatocytes and secreted extracellularly in the uPA-Tg mice. The hepatocytes have small lipid droplets and exhibit growth disorder [3]. On the other hand, uPA is known to digest the extracellular matrix in the liver and trigger the growth of hepatocytes after partial hepatectomy [4] and has a role in activating hepatocyte growth factor [5]. From these results, it is believed that uPA induces engraftment of transplanted hepatocytes and stimulates the growth of the engrafted hepatocytes. The uPA-Tg mice were crossed with immunodeficient mice, nude mice, and were transplanted with rat hepatocytes, resulting in successful rat hepatocyte-chimeric mouse production [6]. Many researchers have been trying to produce chimeric mice whose liver is replaced with human hepatocytes by using host mice with liver disorders and immunodeficiencies. Human liver chimeric mice were generated using uPA/RAG2−/−, uPA/severe combined immunodeficiency (SCID), Fah−/−/Rag2−/−/Il2rg−/− and herpes simplex virus type-1 thymidine kinase-NOG (TK-NOG) mice [7–10]. However, they showed a repopulation index (RI) of 10–70%, and these mice were used for infection studies of hepatitis B viruses (HBV) or hepatitis C viruses (HCV) [7, 8]. We succeeded in producing highly repopulated humanized chimeric mice at an RI of more than 70% stably using uPA/SCID mice (PXB-mouse^®^) [11]. These highly repopulated chimeric mice can be used as a humanized model for not only HBV and HCV infection studies [12, 13], but also for prediction of human metabolism and toxicity [14–19]. However, uPA/SCID mice show four disadvantages: the human hepatocyte RI in mouse liver is decreased due to deletion of the uPA transgene by homologous recombination, kidney disorders are likely to develop, body size is small, and hemizygotes cannot be used as hosts as they undergo more frequent homologous recombination than homozygotes. To correct for these disadvantages, we have established a novel host strain that has a transgene containing albumin promoter/enhancer-driven urokinase-type plasminogen activator cDNA and has a SCID background (cDNA-uPA/SCID) [20]. We succeeded in generating chimeric mice using the hemizygote cDNA-uPA/SCID mice (PXB-mouse^®^), which showed constant increase of body weight and constant increase in human hepatocyte RI since there was no deletion of uPA genes and no kidney disorders. Furthermore, like uPA/SCID chimeric mice, hemizygous cDNA-uPA/SCID chimeric mice were successfully infected with HBV and HCV. These results indicate that hemizygous cDNA-uPA/SCID mice may be useful hosts for producing chimeric mice for use in long-term studies, including hepatitis virus infection analysis or drug toxicity studies [20].

## Characteristics of PXB-mice

**Fig. 1:**
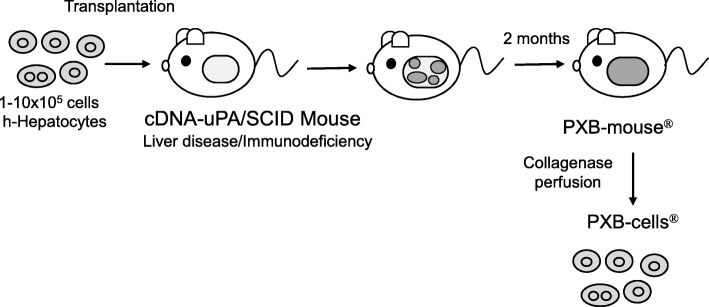
Scheme for production of PXB-mice and PXB-cells

**Fig. 2:**
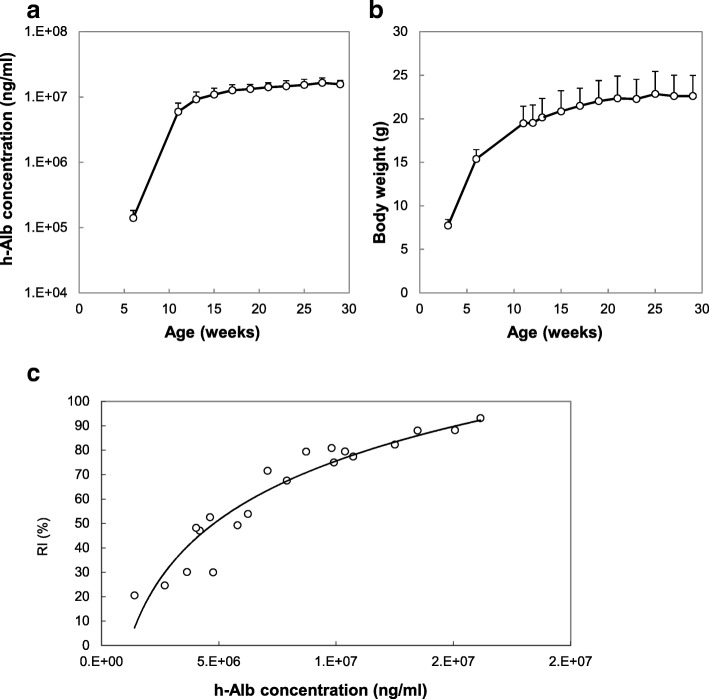
Changes in h-alb levels and body weight of PXB-mice and correlation between h-alb and RI. 14-week-old chimeric mice with > 70% RI were selected based on h-alb levels and observed until they were 28 or 29 weeks old. **a** h-alb levels in chimeric mice increased gradually until mice were at least 28 weeks old. **b** Body weight of increased in chimeras until mice were 18 weeks old, and then stabilized. **c** RI and h-alb concentrations plots at 17-week-old showed correlation

**Fig. 3:**
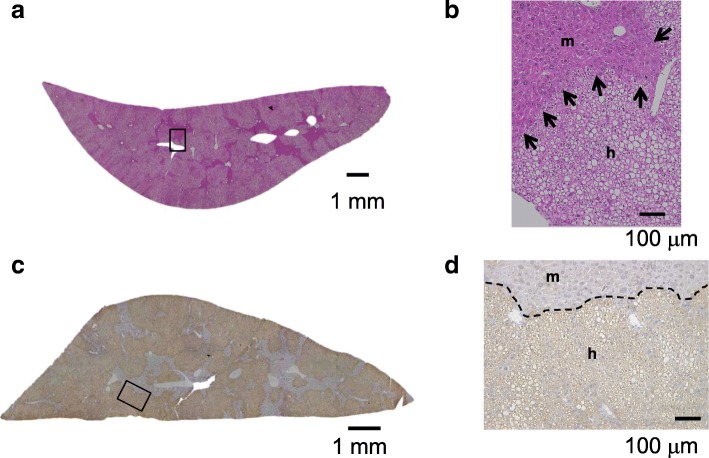
Histological findings in PXB-mouse livers. **a** H&E staining of left lateral lobes. Area within rectangle is magnified in (**b**). Human hepatocytes with clear cytoplasm and lipid droplets occupied most areas of the liver section. **b** Mouse hepatocytes with eosinophilic cytoplasm of various sizes are shown by arrows. **c** The left lateral lobe of PXB-mouse was immunostained with anti-hCK8/18 antibodies. Human hepatocytes were brown-colored, and the area within the rectangle is magnified in (**d**). **d** m, mouse hepatocytes, h, human hepatocytes. H&E staining of 14 weeks. Bar = 100 μm

Cryopreserved human hepatocytes (1–10 × 10^5^ cells) were transplanted into 2–4-week-old hemizygous cDNA-uPA/SCID mice via spleen. Transplanted human hepatocytes engrafted and grew in the host mouse liver, and at 2 months after transplantation we obtained chimeric PXB-mice (Fig. 1). Blood human albumin (h-alb) levels and body weight gradually increased in the hemizygous cDNA-uPA/SCID mice and then were maintained until they were approximately 30 weeks old (Fig. 2a, b). h-Alb levels in mouse blood were well correlated with human hepatocyte RI of the mouse liver (Fig. 2c). H&E stained sections of hemizygote cDNA-uPA/SCID chimeric mouse livers showed that area most occupied with human hepatocytes had clear cytoplasm, and various-sized mouse hepatocytes with eosinophilic cytoplasm were observed (Fig. 3a, b). The RI was calculated as the ratio of the area occupied by human cytokeratin 8/18 (hCK8/18)-positive human hepatocytes to the entire area examined on immunohistochemical sections from seven lobes of the liver (Fig. 3c, d) [11, 20, 21].

**Fig. 4:**
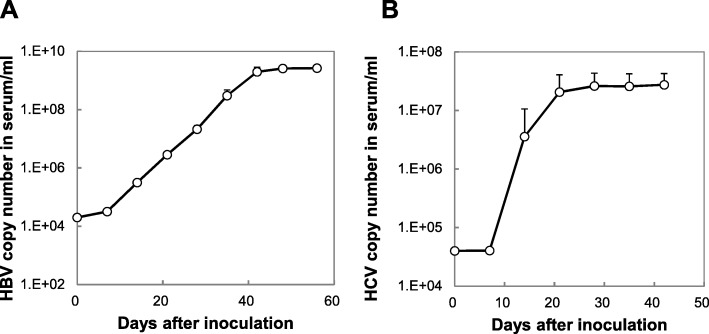
HBV and HCV infections in PXB-mice. PXB-mice were inoculated with **a** HBV and **b** HCV, and copies of HBV and HCV in serum were monitored up to 70 days and 40 days post-infection, respectively

PXB-mice (14–15 weeks old) were infected intravenously with 10^4^ copies of HBV or HCV. Copies of HBV DNA and HCV RNA increased and reached a plateau 40–50 days and 28 days after inoculation, respectively, with HCV RNA copies increasing more rapidly than those of HBV (Fig. 4) [20].

Gene expression levels were compared between hepatocytes from PXB-mouse livers (uPA/SCID mice) and hepatocytes from human livers by microarray analysis. As a result, 82% of transcripts were expressed in both hepatocytes within a 2-fold range difference. The expression data of hepatocyte genes from PXB-mice, human hepatocytes from human liver tissues, and other organs or tissues can be searched at this URL (http://www.ncbi.nlm.nih.gov/sites/GDSbrowser?acc=GDS4327) [22]. Gene expression levels of the liver were similar between hepatocytes from uPA/SCID-chimeric mice and cDNA-uPA/SCID-chimeric mice by microarray analysis [20].

A bovine growth hormone (GH)-transgenic mouse study revealed that the binding of GH to the growth hormone receptor (GHR) increased the expression of IGF-1, a downstream gene of GHR, and decreased expression of lipogenic genes such as FASN and SCID [23]. We reported that since mouse GH (mGH) secreted from the mouse pituitary cannot bind to human GHR (hGHR) due to species differences (amino acid homology of 66%), mRNA expression levels of insulin growth factor (IGF)-1 were downregulated and FASN, FDS1, and SCD1 mRNAs were upregulated in human hepatocytes in the PXB-mouse liver, resulting in accumulation of lipid in the liver (Fig. 3b). Treating PXB-mice with human growth hormone (hGH) improved the fatty liver [24]. Adult-onset GH deficiency (AGHD) is a nonalcoholic fatty liver disease (NAFLD), and GH administration drastically improved the fat accumulation in the liver [25]. These mice will be useful for investigating the mechanism of the action of hGH on human hepatocytes in vivo and the role of GH in AGDH and NAFLD/non-alcoholic steatohepatitis (NASH).

In the mouse body, mouse fibroblast growth factor 15 (mFGF15) is secreted from the small intestine and binds to mouse fibroblast growth factor receptor 4 (mFGFR4) on mouse hepatocytes, and suppresses mouse cytochrome P450 (CYP)7A1, a downstream gene of mFGFR4 that produces bile acid. In humans, human FGF19 (hFGF19), a homolog protein of FGF15, is secreted from small intestine. Naugler et al. reported that in human hepatocyte-chimeric mice (FRG mice), mFGF15 secreted from the mouse intestine cannot bind to human FGFR4 (hFGFR4), resulting in a high concentration of bile acid in mouse serum due to human CYP7A1 induction. Since some bile acid induces hepatocyte growth, liver to body weight ratio was increased in chimeric mice [26]. Incompatibilities between mouse cytokines or hormones derived from mice and human receptors on human hepatocytes in the chimeric mice might exist beyond GH and FGF15.

## Fresh human hepatocytes isolated from PXB-mice (PXB-cells)

Usually, frozen pediatric hepatocytes (6-month-old to 14-years-old) are used as donor cells for chimeric mice because younger hepatocytes have superior growth after transplantation than older hepatocytes [21]. Human hepatocytes (1–10 × 10^5^ cells) are transplanted into cDNA-uPA/SCID mice between 2 and 4 weeks of age. We can isolate 1–2 × 10^8^ hepatocytes from a 12–20-week old PXB-mouse liver by the two-step collagenase perfusion method. We call the hepatocytes PXB-cells^®^. The human hepatocytes proliferated up to 2000-fold in the mouse liver from transplantation to isolation.

Fresh human hepatocytes are known to be the most useful cells for in vitro study of metabolism and chemical toxicity [27]. However, it is difficult to obtain fresh human hepatocytes on demand, and reproducible studies using the same donor cells are impossible. Therefore, researchers in many pharmaceutical companies and in academia have been utilizing cryopreserved human hepatocytes to study metabolism, CYP induction, or hepatocyte toxicity in vitro. However, viability, function, and plating ability vary among the different types of cryopreserved hepatocytes, and the number of vials is limited (100–400 vials). In contrast, we can provide fresh human hepatocytes from the same donor on demand for at least 5 years. Hepatocytes isolated from PXB-mice contain about 5–10% mouse cells (nonparenchymal cells and mouse hepatocytes in a 50/50 ratio), contaminated mouse cells decrease gradually during culture. One of the prominent characteristics of PXB-cells is their superior plating ability. They can be cultured confluently for at least 21 days. They show high mRNA levels of CYPs, UDP-glucuronosyltransferases (UGTs), and transporters (data not yet published) and PXB-cells are susceptible to HBV [28].

When five genome equivalent copies of HBV per cell were infected into PXB-cells, more than 10^6^ copies/mL of HBV DNA were detected in the supernatant of medium, and about 80% of PXB-cells were hepatitis B surface antigen (HBsAg)-positive at 32 days and cccDNA levels increased twice at 7 to 12 days after infection. Lamivudine and hepatitis B immune globulin (HBIG) treatment reduced HBV DNA. The PXB-cells have been used for in vitro study of new anti-HBV drugs [28].

## Applications using chimeric mice for drug development or liver toxicity

By October 2019, a total of 203 papers utilizing several types of chimeric mice or PXB-cells were published describing efficacy studies on HBV or HCV agents, or drug metabolism and pharmacokinetics (DMPK) studies including absorption, distribution, metabolism, and excretion (ADME), drug-drug interaction (DDI), and liver toxicity studies using drugs or chemicals. Papers on studies that did not use chemicals or drugs are not included in this number. We categorized HBV and HCV-efficacy studies by type of entity studied, and also categorized DMPK studies into ADME and DDI studies. FRG mice, TK-NOG mice, and uPA/SCID mice from KMT Hepatech, University Medical Center Hamburg-Eppendorf, Ghent University, and Inserm were used as chimeric mice with humanized livers in addition to PXB-mice (Table 1).

**Table 1:** Publications using humanized mouse/cells

Chimeric mice or cells	PXB-mouse	FRG mouse	TK-NOG mouse	uPA/SCID mouse (CA)^a^	uPA/SCID mouse (DE)^b^	uPA/SCID mouse (BE)^c^	uPA/SCID mouse (FR)^d^	PXB-cells	Total
Anti-HBV agents									
Nucleic acid analog	2	0	0	0	2	0	0	1	5
Small molecule	7	1	0	0	0	0	0	10	18
Interferon	3	0	0	0	3	0	0	0	6
siRNA/miRNA	2	1	1	0	0	0	0	2	6
Antibody	1	1	0	0	0	0	0	0	2
Others	0	1	0	0	6	0	0	0	7
Ant-HCV agents									
Small molecule	24	3	2	3	0	4	1	0	37
Interferon	5	0	0	2	1	0	0	0	8
Small molecule +Interferon	4	0	1	0	0	0	0	0	5
siRNA	4	1	0	0	0	0	0	0	5
Antibody	3	3	0	3	1	9	0	0	19
Others	2	0	0	0	0	0	0	0	2
DMPK/Toxicology									
ADME	24	3	11	0	0	10	0	2	50
DDI	5	0	3	0	0	0	0	1	9
Liver toxicity	19	0	5	0	0	0	0	0	24
Total	105	14	23	8	13	23	1	16	203

^a^ KMT Hepatech, ^b^ University Medical Center Hamburg-Eppendorf, ^c^ Ghent University, ^d^ Inserm

**Fig. 5:**
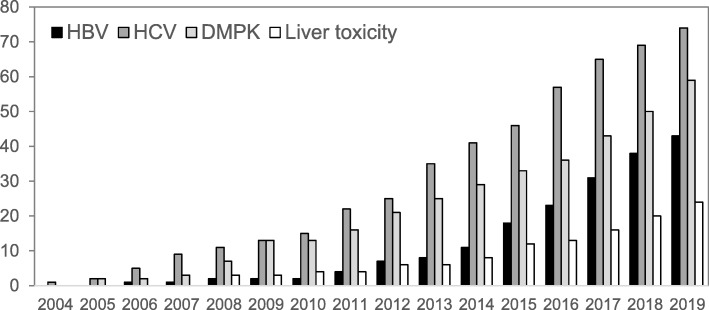
Cumulative number of papers investigating liver-humanized chimeric mice with agents or chemicals

Although the livers of humans and chimpanzees are generally susceptible to HBV or HCV, recently, usage of chimpanzees has been restricted by animal welfare laws. As a result, other small animal models or in vitro culture models are required. Dandri et al. first reported partial repopulation of the liver of uPA/RAG2−/− mice with human hepatocytes. Inoculation of the chimeric mice with HBV led to the establishment of productive HBV infection. Human hepatocytes repopulated up to 15% of the uPA/RAG2−/− mouse liver [7]. HBV infection models were established using PXB-mice, FRG mice, TK-NOG mice, and uPA/SCID mice at University Medical Center Hamburg-Eppendorf. There were publications on efficacy studies of small molecules, interferon, and siRNA in addition to nucleic acid analogs using chimeric mice (Table 1, Additional file 1: Table S1). There were only a few HBV-related papers between 2006 to 2011, but after 2012, the number of publications drastically increased. In addition, from 2014 to the present, 13 papers using PXB-cells were published on HBV-efficacy studies. Due to the high infectivity and high stability of HBV in PXB-cells, the PXB-cells should be suitable for in vitro efficacy studies of new agents against HBV. We expect that the number of HBV-related publications will increase and may surpass the number of HCV-related publications after a few years (Fig. 5).

Marcel et al. first reported that they established an HCV-infected mouse model using uPA/SCID chimeric mice [8]. The number of publications using chimeric mice with anti-HCV agents has gradually increased since 2004, and from 2013 to 2016, the number of publications increased greatly. After 2016, the rate of publication diminished because direct-acting antiviral (DAA) therapy, an effective treatment for HBV, was found. From 2015 to the present, 18 papers with DAAs using chimeric mice were published out of a total of 76 HCV-related papers in which the efficacy of DAA or DAA/interferon combinations were investigated. Excluding small molecules, five papers on siRNA and 19 papers on antibodies were also published (Table 1, Additional file 2: Table S2).

mRNA expression levels of five isozymes of CYPs (CYP1A1, 1A2, 2C9, 2C19, 3A4) were compared between donor livers and PXB-mouse livers, and we demonstrated that the expression levels of these 5 CYPs were very similar [11]. Using LC-MS/MS, protein expression levels of CYP, UGT, and transporters were also compared between ten human livers and six PXB-mouse livers transplanted with two donors. These protein expression levels were all within a 4-fold range difference [29]. From these results, researchers investigated whether humanized chimeric mice can be used for the prediction of human type DMPK. As a result, human type metabolism was reproduced in chimeric mice, but some human-specific metabolites were not detected for some chemicals (SUN13834, diisononyl phthalate, benzydamine) (Additional file 3: Table S3). The remaining mouse hepatocytes might be responsible for the discrepancies in some cases. To improve prediction of drug metabolism and pharmacokinetics in humans, we developed murine Cyp3a−/− chimeric mice with humanized livers, whose livers and small intestines do not express murine Cyp3a [30]. A metabolic profile study of nefazodone-treatment in the Cyp3a−/− chimeric mice demonstrated that Cyp3a−/− chimeric mice may be useful in predicting the metabolic profiles of drug candidates that are extensively metabolized by mouse Cyp3a [31].

Sanoh et al. reported that the relationships of both total clearance (CLt) and the volume of distribution at steady state (Vdss) between those predicted by single-species allometric scaling (SSS) of PXB-mice and the observed human values indicated a good correlation for 17 drugs metabolized by CYPs and non-CYPs [32]. Furthermore, CLt and Vdss values in PXB-mice, monkeys, and rats were determined following intravenous administration of 30 compounds known to be eliminated in humans mainly via hepatic metabolism by various drug-metabolizing enzymes. PXB-mice showed a higher predictability for CLt and Vdss values than the other animal models [33]. These results demonstrate the utility of PXB-mice in predicting human Pharmacokinetics parameters.

Metabolic enzyme induction is a side effect of some drugs, and it can cause serious problems with drug metabolism and toxicity, including reduction of a drug’s effect and an increase in reactive metabolites. Induction of human CYP using PXB-mice and PXB-cells, which were derived from the same donor, was performed. PXB-mice and PXB-cells were treated with 3-methylcholanthrene (3-MC) or rifampicin. Using PXB-mice and PXB-cells, induction levels can be compared between in vivo and in vitro experiments using the same donor cells [34].

Generally, liver damage is determined using hepatotoxicity markers like alanine aminotransferase (ALT) or aspartate transaminase (AST). However, more than 70% of PXB-mice hepatocytes are human hepatocytes, while less than 30% are mouse hepatocytes. When ALT or AST increased in PXB-mouse serum after treatment with a compound, we could not distinguish whether human or mouse hepatocytes or both were damaged by the compound. To solve this problem, we have developed a human ALT1 ELISA kit (data not yet published). Using the ELISA kit, we can detect human hepatocyte-specific damage in toxicological studies of chimeric mice.

Genotoxicity assays are composed of a method, an in vitro or in vivo target, and an endpoint. Although many cell types have been used as targets, human cells are the most important target for evaluating the risk to humans associated with exposure to chemicals. We employed a single-cell gel electrophoresis (comet) assay to detect DNA damage and a micronucleus assay using PXB-mice to evaluate chromosomal aberrations. Treatment with N-ethyl-N-nitrosourea (ENU) increased the percentage of tail DNA in the comet assay in the chimeric mice using uPA/SCID or cDNA-uPA/SCID mice. A liver micronucleus (MN) assay was conducted on the chimeric mice. The number of micronucleated hepatocytes significantly increased. In the bone marrow, the MN assay showed a positive response to ENU in the chimeric mice. Although aflatoxin B1 (AFB1) has strong toxic and carcinogenic effects in the liver, in this study, treatment with AFB1 failed to yield a positive response in the liver comet assay. Although more studies are needed to establish chimeric animal models for human risk assessment, PXB-mice can serve as a valuable model system for genotoxicity assays [35].

Papers about DMPK using chimeric mice began appearing in publications in 2005 and increased gradually, and now the total number of DMPK-related papers is between the total number of HBV and HCV-related papers. Liver toxicology papers have been published since 2008 and also gradually increased, and are about half the number of DMPK-related papers (Fig. 5, Table 1, and Additional file 3: Table S3).

## Discussion

Many researchers have been trying to develop chimeric mice with human hepatocytes since Rhim et al. reported that the liver of uPA-Tg mice repopulated with normal mouse hepatocytes [2]. Although many researchers reported development of humanized chimeric mice with human hepatocytes that have a repopulation ratio of less than 50%, it was challenging to stably produce chimeric mice with more than 70% human hepatocytes. We succeeded in producing chimeric mice whose livers are repopulated with human hepatocytes at 70% and the production ratio was more than 75% [11, 20]. Stable, mass production is essential to be able to utilize humanized chimeric mice as an experimental animal model. Phenomena that occurr in this xenotransplantation model can reveal biological and disease mechanisms by showing the incompatibility in host mice and receptors to human hepatocytes due to species differences between cytokines or hormones in the chimeric mice, such as the incompatibility of mGH with hGHR, or mFGF15 with hFGFR4.

Species differences are known to exist in metabolism and toxicology in drug development. Liver toxicity was found in humans for drugs like troglitazone, and the proportion of drugs that is successful is still low [36, 37]. In the past decade, chimeric mice with human hepatocytes have been produced by several methods, and these chimeric mice have been employed as humanized models for drug development in metabolism and efficacy studies of HBV, HCV, and malaria, and for liver toxicity studies.

Effective anti-HCV drugs like DAA have been launched, and HCV can be eliminated in many patients by DAA treatment. Presently, development of anti-HBV agents is being actively pursued using chimeric mice with humanized livers. NAFLD has emerged as the foremost chronic liver disease within the past two decades in Western countries. Some NAFLD patients are likely to develop NASH, a progressive liver disease, which can lead to liver fibrosis, cirrhosis, and hepatocellular carcinoma. Human NASH models are essential for assessing the efficacy and safety of new drugs developed for treating this condition. We are now developing NASH models using the chimeric mice.

Although HBV- and HCV-models using chimeric mice are useful as a persistent infection model to investigate efficacy of agents that fight viruses, hepatitis and fibrosis do not occur in the chimeric mice due to immunodeficiency. Recently, dual chimeric mice with human hepatocytes and components of a human immune system were developed [38, 39]. Not only do the dual chimeric mice have human hepatocytes and immune cells, if chimeric mice were to be developed with human microorganisms, kidneys, pancreas, or gut, a humanized model with more accurate human physiology could be created that would be useful to develop new drugs that are safe and more effective.

## Conclusions

We conclude that PXB-mouse livers show nearly normal morphology and express most genes at levels similar to those expressed by normal human livers. PXB-mouse livers will therefore be useful for long-term studies, including those on hepatitis virus infection and drug toxicity. Chimeric mice with humanized livers and human hepatocytes isolated from these mice have immense potential in future applications, such as for studies on efficacy and safety, as a humanized model to support the development of new drugs.

## Supplementary information

**Additional file 1: Table S1.** Publications on HBV using humanized mouse/cells.

**Additional file 2: Table S2.** Publications on HCV using humanized mouse.

**Additional file 3: Table S3.** Publications on DMPK and liver toxicity using humanized mouse/cells.

## Data Availability

The dataset supporting the conclusions of this article is available in the NCBI Gene Expression Omnibus (GEO) repository, http://www.ncbi.nlm.nih.gov/sites/GDSbrowser?acc=GDS4327.

## Abbreviations

3-MC: 3-methylcholanthrene
ADME: Absorption, distribution, metabolism, and excretion
AFB1: Afratoxin B1
AGHD: Adult-onset GH deficiency
ALT: Alanine aminotransferase
AST: Aspartate transaminase
CLt: Total clearance
CYP: Cytochrome P450
DDI: Drug-drug interaction
DMPK: Drug metabolism and pharmacokinetics
ENU: N-ethyl-N-nitrosourea
FGF15: Fibroblast growth factor 15
FGFR4: Fibroblast growth factor receptor 4
GH: Growth hormone
GHR: Growth hormone receptor
h-alb: Human albumin
HBIG: Hepatitis B immune globulin
HBsAg: Hepatitis B surface antigen
HBV: Hepatitis B viruses
hCK8/18: Human cytokeratin 8/18
HCV: Hepatitis C viruses
hGH: Human growth hormone
hGHR: Human growth hormone receptor
IGF: Insulin growth factor
mGH: Mouse growth hormone
MN: Micronucleus
NAFLD: Non-alcoholic fatty liver disease
NASH: Non-alcoholic steatohepatitis
PK: Parmacokinetics
RI: Repopulation index
SCID: Severe combined immunodeficiency
SSS: Single-species allometric scaling
TK-NOG: Herpes simplex virus type-1 thymidine kinase-NOG
UGT: UDP-glucuronosyltransferase
uPA-Tg: Urokinase plasminogen activator transgenic
Vdss: Volume of distribution at steady state
