# Supplementary material for: Characterization and applications of chimeric mice with humanized livers for preclinical drug development
Source: Lab Anim Res. 2020 Jan 8;36:2. doi: 10.1186/s42826-019-0032-y (PMC7081693; doi:10.1186/s42826-019-0032-y)
Supplement: Supplementary file 1 — Additional file 1: Table S1. Publications on HBV using humanized mouse/cells. [file 42826_2019_32_MOESM1_ESM.pdf]

Supplementary Table 1. Publications on HBV using humanized mouse/cells

Category	No. of papers	Agents	PXB-mouse	FRG mouse	TK-NOG mouse	uPA/SCID mouse (DE)*	PXB-cells	Publications
Nucleic acid analog	5	Lamivudine & Myrcludex-B				○		Allweiss L, et al. Proliferation of primary human hepatocytes and prevention of hepatitis B virus reinfection efficiently deplete nuclear cccDNA in vivo. Gut. 2018 Mar;67(3):542-552.
		Tenofovir DF, M3	○					Murakami E, et al. Effect of tenofovir disoproxil fumarate on drug-resistant HBV clones. J Infect. 2016 Jan;72(1):91-102.
		HBV-active NAs, LAM, TDF					○	Watanabe T, et al. Postexposure Prophylactic Effect of Hepatitis B Virus (HBV)-Active Antiretroviral Therapy against HBV Infection. Antimicrob Agents Chemother. 2015 Feb;59(2):1292-1298.
		l-Hyd4FC				○		T Volz, et al. Strong antiviral activity of the new l-hydroxycytidine derivative, l-Hyd4FC, in HBV-infected human chimeric uPA/SCID mice . Antivir Ther. 2012;17(4):623-631.
		Lamivudine	○					Tsuge M, et al. Infection of human hepatocyte chimeric mouse with genetically engineered hepatitis B virus. Hepatology. 2005 Nov;42(5):1046-1054.
Small molecules	18	ARB-1740	○					Ye X, et al. Hepatitis B Virus Therapeutic Agent ARB-1740 Has Inhibitory Effect on Hepatitis Delta Virus in a New Dually-Infected Humanized Mouse Model. ACS Infect Dis. 2019 May 10;5(5):738-749.
		ARB-1740	○					Thi EP, et al. ARB-1740, a RNA Interference Therapeutic for Chronic Hepatitis B Infection. ACS Infect Dis. 2019 May 10;5(5):725-737.
		Ciclopirox	○					Kang JA, et al. Ciclopirox inhibits Hepatitis B Virus secretion by blocking capsid assembly. Nat Commun. 2019 May 16;10(1):2184.
		Accutane	○					Birkus G, et al. Anti-HBV activity of retinoid drugs in vitro versus in vivo. Antiviral Res. 2019 Sep;169:104538.
		CDM-3008					○	Furutani Y, et al. An interferon-like small chemical compound CDM-3008 suppresses hepatitis B virus through induction of interferon-stimulated genes. PLoS One. 2019 Jun 12;14(6):e0216139.
		NVR 3-778	○					Klumpp K, at al. Efficacy of NVR 3-778, Alone and in Combination With Pegylated Interferon, vs Entecavir in uPA/SCID Mice With Humanized Livers and HBV Infection. Gastroenterology. 2018 Feb;154(3):652-662.e8.
		Silibinin					○	Umetsu T, et al. Inhibitory effect of silibinin on hepatitis B virus entry. Biochem Biophys Rep. 2018 Mar 31;14:20-25.
		Rosmarinic acid					○	Tsukamoto Y, et al. Rosmarinic acid is a novel inhibitor for Hepatitis B virus replication targeting viral epsilon RNA-polymerase interaction. PLoS One. 2018 May 21;13(5):e0197664.
		Fasiglifam					○	Nio Y, et al. Inhibitory effect of fasiglifam on hepatitis B virus infections through suppression of the sodium taurocholate cotransporting polypeptide. Biochem Biophys Res Commun. 2018 Jun 27;501(3):820-825.
		RG7834	○					Mueller H, et al. A Novel Orally Available Small Molecule That Inhibits Hepatitis B Virus Expression. J Hepatol. 2017 Oct 24.
		proanthocyanidin and its Ananalogs					○	Tsukuda S, et al. A new class of hepatitis B and D virus entry inhibitors, proanthocyanidin and its analogs, that directly act on the viral large surface proteins. Hepatology. 2017 Apr;65(4):1104-1116.

		SCY806, SCY446, SCY450, SCY453, SCY995					○	Shimura S, et al. Cyclosporin derivatives inhibit hepatitis B virus entry without interfering with NTCP transporter activity. J Hepatol. 2017 Apr;66(4):685-692.
		HBV RNaseH inhibitors #110 , #208		○				LongKR, et al. Efficacy of hepatitis B virus ribonuclease H inhibitors, a new class of replication antagonists, in FRG human liver chimeric mice (FRG). Antiviral Res. 2017 Nov 10;149:41-47.
		GW4869 & Anti-CD-81					○	Sanada T, et al. Transmission of HBV DNA Mediated by Ceramide-Triggered Extracellular Vesicles. Cell Mol Gastroenterol Hepatol. 2016 Oct 24;3(2):272-283.
		Ed4T or BMS-986001	○					Takamatsu Y, et al. 4'-modified nucleoside analogs: potent inhibitors active against entecavir-resistant hepatitis B virus. Hepatology. 2015 Oct;62(4):1024-36. doi: 10.1002/hep.27962.
		M11, M12, M13, M17, M18, M19					○	Murakami Y, et al. Discovering novel direct acting antiviral agents for HBV using in silico screening. Biochem Biophys Res Commun. 2015 Jan 2;456(1):20-28.
		Vanitaracin A					○	Kaneko M, et al. Entry of Hepatitis B and D Viruses by Targeting Sodium Taurocholate Cotransporting Polypeptide. J Virol. 2015 Dec;89(23):11945-11953.
		Cyclosporin A and its analogs					○	Watashi K, et al. Cyclosporin A and its analogs inhibit hepatitis B virus entry into cultured hepatocytes through targeting a membrane transporter, sodium taurocholate cotransporting polypeptide (NTCP). Hepatology. 2014 May;59(5):1726-37.
Interferon	6	PEG-IFN Plus ETV	○					Uchida T, et al. Persistent Loss of Hepatitis B Virus Markers in Serum without Cellular Immunity by Combination of Peginterferon and Entecavir Therapy in Humanized Mice. Antimicrob Agents Chemother. 2017 Aug 24;61(9) pii: e00725-17.
		TRK-560	○					Tsuge M, et al. Chayama. Development of a Novel Site-Specific Pegylated Interferon Beta for Antiviral Therapy of Chronic Hepatitis B Virus. Antimicrob Agents Chemother. 2017 May 24;61(6) e00183-17.
		PegIFNalpha				○		Allweiss L, et al. Immune cell responses are not required to induce substantial hepatitis B virus antigen decline during pegylated interferon-alpha administration. J Hepatol. 2014 Mar;60(3): 500-7.
		IFN-alpha				○		Belloni L, et al. IFN-alpha inhibits HBV transcription and replication in cell culture and in humanized mice by targeting the epigenetic regulation of the nuclear cccDNA minichromosome. J Clin Invest. 2012 Feb 1;122(2):529-537.
		IFN-α	○					Tsuge M, et al. Effects of hepatitis B virus infection on the interferon response in immunodeficient human hepatocyte chimeric mice. J Infect Dis. 2011 Jul 15;204(2):224-8.
		IFN-alpha				○		Lutgehetmann M, et al. Hepatitis B virus limits response of human hepatocytes to interferon-alpha in chimeric mice. Gastroenterology. 2011 Jun;140(7):2074-83, 2083.e1-2.
siRNA/miRNA	6	GalNAc-LNPs	○					Sato Y, et al. Highly specific delivery of siRNA to hepatocytes circumvents endothelial cell-mediated lipid nanoparticle-associated toxicity leading to the safe and efficacious decrease in the hepatitis B virus. J Control Release. 2017 Oct 3;266:216-225.
		HBV-siRNAmix					○	Yamamoto N, et al. Novel pH-sensitive multifunctional envelope-type nanodevice for siRNA-based treatments for chronic HBV infection. J Hepatology 2016; 64: 547–555.
		NTPC-siRNA			○			Nakabori T, et al. Sodium taurocholate cotransporting polypeptide inhibition efficiently blocks hepatitis B virus spread in mice with a humanized liver. Sci Rep. 2016 Jun 9;6:27782. doi: 10.1038/srep27782.
		siHBV, siNTCP, siLTC					○	Ishida Y, et al. Novel robust in vitro hepatitis B virus infection model using fresh human hepatocytes isolated from humanized mice. Am J Pathol. 2015 May;185(5):1275-85.
		HBV-siRNA		○				Shih YM, et al. Combinatorial RNA interference therapy prevents selection of pre-existing HBV variants in human liver chimeric mice. Sci Rep. 2015 Oct 20;5:15259.

		hsa-miR-1231	○					Kohno T, et al. Human microRNA hsa-miR-1231 suppresses hepatitis B virus replication by targeting core mRNA. J Viral Hepat. 2014 Sep;21(9):e89-97.
Antibody	2	mAb E6F6		○				Zhang TY, et al. Prolonged suppression of HBV in mice by a novel antibody that targets a unique epitope on hepatitis B surface antigen. Gut. 2016 Apr;65(4):658-671.
		Antibody HB0116 and HB0478	○					Hamada-Tsutsumi S, et al. Validation of cross-genotype neutralization by hepatitis B virus-specific monoclonal antibodies by in vitro and in vivo infection. PLoS One. 2015 Feb 18;10(2):e0118062.
Others	7	TCR-grafted T cells				○		Wisskirchen K, et al. T cell receptor grafting allows virological control of Hepatitis B virus infection. J Clin Invest. 2019 Apr 30;129(7):2932-2945.
		HBV-TCR RE-T cells				○		Koh S, et al. Non-lytic Lymphocytes Engineered to Express Virus-specific T-cell Receptors Limit HBV Infection by Activating APOBEC3. Gastroenterology. 2018 Jul;155(1):180-193.e6.
		HBsAg-redirected T cells		○				Kruse R, et al. HBsAg-redirected T cells exhibit antiviral activity in HBV-infected human liver chimeric mice. Cytotherapy. 2018 May;20(5):697-705.
		TCR-T cells				○		Kah J, et al. Lymphocytes transiently expressing virus-specific T cell receptors reduce hepatitis B virus infection. J Clin Invest. 2017 Aug 1;127(8):3177-3188.
		Myrcludex-B				○		Volz T, et al. The entry inhibitor Myrcludex-B efficiently blocks intrahepatic virus spreading in humanized mice previously infected with hepatitis B virus. J Hepatol. 2013 May;58(5):861-867.
		Myrcludex-B				○		Lutgehetmann M, et al. Humanized chimeric uPA mouse model for the study of hepatitis B and D virus interactions and preclinical drug evaluation. Hepatology. 2012 Mar;55(3):685-694.
		HBV preS/2-39^{myr} and 48^{stearoyl}				○		Petersen J, et al. Prevention of hepatitis B virus infection in vivo by entry inhibitors derived from the large envelope protein. Nat Biotechnol. 2008 Mar;26(3):335-341.

*, University Medical Center Hamburg-Eppendorf
