# Supplementary material for: Characterization and applications of chimeric mice with humanized livers for preclinical drug development
Source: Lab Anim Res. 2020 Jan 8;36:2. doi: 10.1186/s42826-019-0032-y (PMC7081693; doi:10.1186/s42826-019-0032-y)
Supplement: Supplementary file 2 — Additional file 2: Table S2. Publications on HCV using humanized mouse. [file 42826_2019_32_MOESM2_ESM.pdf]

Supplementary Table 2. Publications on HCV using humanized mouse

Category	No. of papers	Agents	PXB-mouse	uPA/SCID mouse (CA)*[1]	FRG mouse	TK-NOG mouse	uPA/SCID mouse (BE)*[2]	uPA/SCID mouse (DE)*[3]	uPA/SCID moue (FR)*[4]	Publications
Small molecules	37	Daclatasvir plus Asunaprevir; GLE/PIB	O							Osawa M, et al. Efficacy of glecaprevir and pibrentasvir treatment for genotype 1b hepatitis C virus drug resistance-associated variants in humanized miceJ Gen Virol. 2019 Jul;100(7):1123-1131.
		MK-7009, BMS-788329 DAAs	O							Hamdane N, et al. HCV-induced epigenetic changes associated with liver cancer risk persist after sustained virologic response. Gastroenterology. 2019 Jun;156(8):2313-2329.e7.
		Asunaprevir, Beclabuvir, Daclatasvir	O							Teraoka Y, et al. Limitations of daclatasvir/asunaprevir plus beclabuvir treatment in cases of NS5A inhibitor treatment failure. J Gen Virol. 2018 Aug;99(8):1058-1065.
		Glecaprevir+ Pibrentasvir+ Sofosbuvir	O							Teraoka Y, et al. Prevalence of NS5A resistance associated variants in NS5A inhibitor treatment failures and an effective treatment for NS5A-P32 deleted hepatitis C virus in humanized miceBiochem Biophys Res Commun. 2018 Jun 2;500(2):152-157.
		Chlorcyclizine Derivatives	O							Rolt A, et al. Preclinical Pharmacological Development of Chlorcyclizine Derivatives for the Treatment of Hepatitis C Virus Infection. J Infect Dis. 2018 May 5;217(11):1761-1769.
		Daclatasvir, Asunaprevir , Beclabuvir			O					Burchill MA, et al. Rapid reversal of innate immune dysregulation in blood of patients and livers of humanized mice with HCV following DAA therapy. PLoS One. 2017 Oct 17;12(10):e0186213.
		Corilagin	O							Reddy BU, et al. A natural small molecule inhibitor corilagin blocks HCV replication and modulates oxidative stress to reduce liver damage. Antiviral Res. 2017 Dec 7;150:47-59.
		BI-2536			O					Diab A, et al. Polo-like-kinase 1 is a proviral host-factor for hepatitis B virus replication. Hepatology. 2017 Dec;66(6):1750-1765.
		jumonji domain-containing protein 6				O				Ganesan M, et al. Demethylase JMJD6 as a New Regulator of Interferon Signaling: Effects of HCV and Ethanol Metabolism. Cell Mol Gastroenterol Hepatol. 2017 Oct 16;5(2):101-112.
		Vaniprevir, BMS-788329	O							Uchida T, et al. Elimination of HCV via a non-ISG-mediated mechanism by vaniprevir and BMS-788329 combination therapy in human hepatocyte chimeric mice. Virus Res. 2016 Feb 2;213:62-68
		Daclatasvir Asunaprevir	O							Kan H, et al. Infection of human hepatocyte chimeric mouse with genetically engineered hepatitis B virus. Antivir Ther. 2016;21(4):307-315.
		Legalon SIL	O							DebRoy S, et al. Hepatitis C virus dynamics and cellular gene expression in uPA-SCID chimeric mice with humanized livers during intravenous silibinin monotherapy. J Viral Hepat. 2016 Sep;23(9):708-717.
		B5			O					Vausselin T, et al. Identification of a new benzimidazole derivative as an antiviral against hepatitis C virus. J Virol. 2016 Sep 12;90(19):8422-8434.
		Ledipasvir/GS-558093, Telaprevir/GS-558093				O				Kai Y, et al. Emergence of hepatitis C virus NS5A L31V plus Y93H variant upon treatment failure of daclatasvir and asunaprevir is relatively resistant to ledipasvir and NS5B polymerase nucleotide inhibitor GS-558093 in human hepatocyte chimeric mice. Gastroenterol. 2015 Nov;50(11):1145-1151.
		GSK8853	O							Pouliot JJ, et al. Preclinical characterization and in vivo efficacy of GSK8853, a small molecule inhibitor of the Hepatitis C virus NS4B protein. Antimicrob Agents Chemother. 2015 Oct;59(10):6539-6550.

erlotinib and dasatinib combined with Anti-CD81/anti-CLDN1							O	Xiao F, et al. Synergy of entry inhibitors with direct-acting antivirals uncovers novel combinations for prevention and treatment of hepatitis C. Gut. 2015 Mar;64(3):483-94.
Compound 1a	O							Miller JF, et al. Hepatitis C replication inhibitors that target the viral NS4B protein. J Med Chem. 2014 Mar 13;57(5):2107-2120.
NA808 and telaprevir	O							Katsume A, et al. A serine palmitoyltransferase inhibitor blocks hepatitis C virus replication in human hepatocytes. Gastroenterology. 2013 Oct;145(4):865-73.
2,3-dihydro-5-hydroxy- 2,2-dipentyl-4,6-di- tert-butylbenzofuran	O							Yasui Y, et al. Synthetic lipophilic antioxidant BO-653 suppresses HCV replication. J Med Virol. 2013 Feb;85(2):241-9.
BMS-605339, BMS-788329 or BMS-821095	O							Shi N, et al. Combination therapies with NS5A, NS3 and NS5B inhibitors on different genotypes of hepatitis C virus in human hepatocyte chimeric mice. Gut. 2013 Jul;62(7):1055-61.
SB-431542, LY-364947	O							Sakata K, et al. HCV NS3 protease enhances liver fibrosis via binding to and activating TGF-beta type I receptor. Sci Rep. 2013 Nov 22;3:3243.
LIC-pIC	O							Nakagawa S, et al. Targeted induction of interferon-lambda in humanized chimeric mouse liver abrogates hepatotropic virus infection. PLoS One. 2013;8(3):e59611.
Artemisinin analogues					O			Obeid S, et al. Artemisinin analogues as potent inhibitors of in vitro hepatitis C virus replication. PLoS One. 2013 Dec 11;8(12):e81783.
RO8191 (small-molecule type I IFN receptor agonist)	O							Konishi H, et al. An orally available, small-molecule interferon inhibits viral replication. Sci Rep. 2012;2:259.
Telaprevir and MK-0608	O							Ohara E, et al. J Elimination of hepatitis C virus by short term NS3-4A and NS5B inhibitor combination therapy in human hepatocyte chimeric mice. Hepatol. 2011 May; 54(5): 872-8.
Telaprevir	O							Hiraga N, et al. Rapid emergence of telaprevir resistant hepatitis C virus strain from wildtype clone in vivo. Hepatology. 2011 Sep 2;54(3):781-8.
ME3738	O							Abe H, et al. ME3738 enhances the effect of interferon and inhibits hepatitis C virus replication both in vitro and in vivo. J Hepatol. 2011 Jul;55(1):11-8.
MK-3281					O			Narjes F, et al. Discovery of (7R)-14-cyclohexyl-7-{[2-(dimethylamino)ethyl](methyl) amino}-7,8-dihydro-6H-indolo[1,2-e][1,5]benzoxazocine-11-carboxylic acid (MK-3281), a potent and orally bioavailable finger-loop inhibitor of the hepatitis C virus NS5B polymerase. J Med Chem. 2011 Jan 13;54(1):289-301.
Recombinant griffithsin					O			Meuleman P, et al. Griffithsin has antiviral activity against hepatitis C virus. Antimicrob Agents Chemother. 2011 Nov;55(11):5159-5167.
Telaprevir	O							Kamiya N, et al. Practical evaluation of a mouse with chimeric human liver model for hepatitis C virus infection using an NS3-4A protease inhibitor. J Gen Virol. 2010 Jul;91(Pt 7):1668-1677.
Amphipathic DNA polymers	O							Matsumura T, et al. Amphipathic DNA polymers inhibit hepatitis C virus infection by blocking viral entry. Gastroenterology. 2009 Aug;137(2):673-681.
DEBIO-025	O							Inoue K, et al. Evaluation of a cyclophilin inhibitor in hepatitis C virus-infected chimeric mice in vivo. Hepatology. 2007 Apr;45(4):921-928.
BILN 2061					O			Vanwolleghem T, et al. Ultra-rapid cardiotoxicity of the hepatitis C virus protease inhibitor BILN 2061 in the urokinase-type plasminogen activator mouse. Gastroenterology. 2007 Oct;133(4):1144-55.

		Myriocin	O							Umehara T, et al. Serine palmitoyltransferase inhibitor suppresses HCV replication in a mouse model. Biochem Biophys Res Commun. 2006 Jul 21;346(1):67-73.
		BH3 interacting domain death agonist		O						Hsu EC, et al. CD Richardson. Modified apoptotic molecule (BID) reduces hepatitis C virus infection in mice with chimeric human livers. Nat Biotechnol. 2003 May;21(5):519-525.
Interferon	8	IFN-alpha	O							Miyaki E, et al. Interferon alpha treatment stimulates interferon gamma expression in type I NKT cells and enhances their antiviral effect against hepatitis C virus. PLoS One. 2017 Mar 2;12(3):e0172412.
		IFN-α2, IFN-γ, IFN-λ1	O							Hamana A, et al. Evaluation of antiviral effect of type I, II, and III interferons on direct-acting antiviral-resistant hepatitis C virus. Antiviral Res. 2017 Aug 31;146:130-138.
		biliverdin & PEG-IFNα						O		Kah J, et al. Heme oxygenase-1 polymorphisms can affect HCV replication and treatment responses with different efficacy in humanized mice. Liver Int. 2017 Aug;37(8):1128-1137.
		IFNα-2b		O						Chen R, et al. Intrinsic Viral Factors Are the Dominant Determinants of the Hepatitis C Virus Response to Interferon Alpha Treatment in Chimeric Mice. PLoS One. 2016 Jan 14;11(1):e0147007. doi: 10.1371
		IFN-gamma gene transfer	O							Takahashi Y,et al. Long-Term Elimination of Hepatitis C Virus from Human Hepatocyte Chimeric Mice After Interferon-gamma Gene Transfer. Hum Gene Ther Clin Dev. 2014 Mar;25(1):28-39.
		PEG-IFN-alpha	O							Watanabe T, et al. Hepatitis C virus kinetics by administration of pegylated interferon-alpha in human and chimeric mice carrying human hepatocytes with variants of the IL28B gene. Gut. 2013 Sep;62(9):1340-1346.
		Interferon alpha	O							Hiraga N, et al. Infection of human hepatocyte chimeric mouse with genetically engineered hepatitis C virus and its susceptibility to interferon. FEBS Lett. 2007 May 15;581(10):1983-1987.
		IFN-alpha		O						Kneteman NM, et al. Anti-HCV therapies in chimeric scid-Alb/uPA mice parallel outcomes in human clinical application. Hepatology. 2006 Jun;43(6):1346-1353.
Small molecule+Interferon	5	Ledipasvir /GS-558093, simeprevir / GS-558093 + PEG-IFN				O				Doi A, et al. Combinations of two drugs among NS3/4A inhibitors, NS5B inhibitors and non-selective antiviral agents are effective for hepatitis C virus with NS5A-P32 deletion in humanized-liver mice. J Gastroenterol. 2019 May;54(5):449-458.
		Simeprevir Daclatasvir Asunaprevir +IFN	O							Kan H, et al. Protease inhibitor resistance remains even after mutant strains become undetectable using deep sequencing. J Infect Dis. 2016 Dec 1;214(11):1687-1694.
		Telaprevir and MK-0608 & IFN	O							de Jong YP, et al. Evaluation of combination therapy against hepatitis C virus infection in human liver chimeric mice. J Hepatol. 2011 May;54(5):848-850.
		Ribavirin +IFN	O							Kurbanov F, et al. Positive selection of core 70Q variant genotype 1b hepatitis C virus strains induced by pegylated interferon and ribavirin. J Infect Dis. 2010 Jun 1;201(11):1663-1671.
		Hsp90 inhibitor and PEG-IFN	O							Nakagawa S, et al. Hsp90 inhibitors suppress HCV replication in replicon cells and humanized liver mice. Biochem Biophys Res Commun. 2007 Feb 23;353(4):882-8.
**siRNA**	5	RRM2-siRNA	O							Kitab B, et al. Ribonucleotide reductase M2 promotes RNA replication of hepatitis C virus by protecting NS5B protein from hPLIC1-dependent proteasomal degradation. J Biol Chem. 2019 Apr 12;294(15):5759-5773.
		siRNA target HCV 5'UTR	O							Watanabe T, et al. In vivo therapeutic potential of Dicer-hunting siRNAs targeting infectious hepatitis C virus. Sci Rep. 2014 Apr 23;4:4750.
		LNP- sshRNAs	O							Ma H, et al. Formulated minimal-length synthetic small hairpin RNAs are potent inhibitors of hepatitis C virus in mice with humanized livers. Gastroenterology. 2014 Jan;146(1):63-66.e5.

		LNP- sshRNAs	O							Dallas S, et al. Inhibition of hepatitis C virus in chimeric mice by short synthetic hairpin RNAs: sequence analysis of surviving virus shows added selective pressure of combination therapy. J Virol. 2014 May;88(9):4647-4656.
		thromboxane A2 synthase			O					Abe Y, et al. Combinatorial RNA Interference Therapy Prevents Selection of Pre-existing HBV Variants in Human Liver Chimeric Mice. Gastroenterology. 2013 Sep;145(3):658-667.
**Antibody**	19	Anti-E2 &anti-claudin 1			O					Mailly L, et al. In vivo combination of human anti-envelope glycoprotein E2 and -Claudin-1 monoclonal antibodies for prevention of hepatitis C virus infection . Antiviral Res. 2019 Feb;162:136-141. doi: 10.1016/j.antiviral.2018.12.018.
		claudin-1-specific monoclonal Ab							O	Colpitts CC, et al. Humanisation of a claudin-1-specific monoclonal antibody for clinical prevention and cure of HCV infection without escape. Gut. 2018 Apr;67(4):736-745.
		mAb 2A5					O			Desombere I, et al. A novel neutralizing human monoclonal antibody broadly abrogates hepatitis C virus infection in vitro and in vivoAntiviral Res. 2017 Dec;148:53-64.
		human mAb		O						O'Shea D, et al. Prevention of hepatitis C virus infection using a broad cross-neutralizing monoclonal antibody (AR4A) and epigallocatechin gallate. Liver Transpl. 2016 Mar;22(3):324-332.
		HC84.26		O						Keck Z, et al. Affinity maturation of a broadly neutralizing human monoclonal antibody that prevents acute hepatitis C virus infection in mice. Hepatology. 2016 Dec;64(6):1922-1933.
		mAb E6F6			O					Zhang TY, et al. Prolonged suppression of HBV in mice by a novel antibody that targets a unique epitope on hepatitis B surface antigen. Gut. 2016 Apr;65(4):658-71. doi: 10.1136
		mAb16-71					O			Vercauteren K, et al. Targeting a host-cell entry factor barricades antiviral-resistant HCV variants from on-therapy breakthrough in human-liver mice. Gut. 2016 Dec;65(12):2029-2034.
		mAbs AP33					O			Desombere I, et al. Monoclonal anti-envelope antibody AP33 protects humanized mice against a patient-derived hepatitis C virus challenge. Hepatology. 2016 Apr;63(4):1120-1134.
		anti-human CD81 mAb	O							Ji C, et al. Prevention of hepatitis C virus infection and spread in human liver chimeric mice by an anti-CD81 monoclonal antibody. Hepatology. 2015 Apr;61(4):1136-1144.
		claudin-1-targeting monoclonal Ab						O		Mailly L, et al. Clearance of persistent hepatitis C virus infection in humanized mice using a claudin-1-targeting monoclonal antibody. Nat Biotechnol. 2015 May;33(5):549-554.
		SR-BI mAbs					O			Vercauteren K, et al. Successful anti-scavenger receptor class B type I (SR-BI) monoclonal antibody therapy in humanized mice after challenge with HCV variants with in vitro resistance to SR-BI-targeting agents. Hepatology. 2014 Nov;60(5):1508-1518.
		IgG purified from the sera of HCV-particle-immunized mice	O							Akazawa D, et al. Neutralizing antibodies induced by cell culture-derived hepatitis C virus protect against infection in mice. Gastroenterology. 2013 Aug;145(2):447-455
		SR-BI, mAb16-71					O			Meuleman P, et al. A human monoclonal antibody targeting scavenger receptor class B type I precludes hepatitis C virus infection and viral spread in vitro and in vivo. Hepatology. 2012 Feb;55(2):364-372.
		anti-SR-BI mAbs					O			Lacek K, et al. Novel human SR-BI antibodies prevent infection and dissemination of HCV in vitro and in humanized mice. J Hepatol. 2012 Jul;57(1):17-23.
		H06-antibodies					O			Meuleman P, et al. In vivo evaluation of the cross-genotype neutralizing activity of polyclonal antibodies against hepatitis C virus. Hepatology. 2011 Mar;53(3):755-762.
		Polyclonal IgG					O			Vanwolleghem T, et al. Polyclonal immunoglobulins from a chronic hepatitis C virus patient protect human liver-chimeric mice from infection with a homologous hepatitis C virus strain.Hepatology. 2008 Jun;47(6):1846-1855.

		Anti-CD81					O			Meuleman P, et al. Anti-CD81 antibodies can prevent a hepatitis C virus infection in vivo. Hepatology. 2008 Dec;48(6):1761-1768.
		BILN-2061; HCV371		O						Kneteman NM, et al. Anti-HCV therapies in chimeric scid-Alb/uPA mice parallel outcomes in human clinical application. Hepatology. 2006 Jun;43(6):1346-1353.
		anti-CLDN1 Mabs	O							Fukasawa M, et al. Monoclonal Antibodies against Extracellular Domains of Claudin-1 Block Hepatitis C Virus Infection in a Mouse Model. J Virol. 2015 May 1;89(9):4866-4879.
Others	2	ribonucleotide reductase M2	O							Kitab B, et al. Ribonucleotide reductase M2 promotes RNA replication of hepatitis C virus by protecting NS5B protein from hPLIC1-dependent proteasomal degradation. J Biol Chem. 2019 Apr 12;294(15):5759-5773.
		peroxisomal biogenesis impaired by HCV	O							Lupberger J, et al. Combined Analysis of Metabolomes, Proteomes, and Transcriptomes of HCV-infected Cells and Liver to Identify Pathways Associated With Disease Development. Gastroenterology. 2019 Aug;157(2):537-551.e9.
		Griffithsin, Scytovirin		O						Takebe Y, et al. Antiviral lectins from red and blue-green algae show potent in vitro and in vivo activity against hepatitis C virus. PLoS One. 2013 May 21;8(5):e64449.
		HCV796		O						Kneteman NM, et al. HCV796: A selective nonstructural protein 5B polymerase inhibitor with potent anti-hepatitis C virus activity in vitro, in mice with chimeric human livers, and in humans infected with hepatitis C virus. Hepatology. 2009 Mar;49(3):745-752.

[*1], KMT Hepatech; [*2], Ghent University; [*3], University Medical Center Hamburg-Eppendorf; [*4], Inserm
