# Supplementary material for: Characterization and applications of chimeric mice with humanized livers for preclinical drug development
Source: Lab Anim Res. 2020 Jan 8;36:2. doi: 10.1186/s42826-019-0032-y (PMC7081693; doi:10.1186/s42826-019-0032-y)
Supplement: Supplementary file 3 — Additional file 3: Table S3. Publications on DMPK and liver toxicity using humanized mouse/cells. [file 42826_2019_32_MOESM3_ESM.pdf]

**Supplementary Table 3. Publications on DMPK and liver toxicity using humanized mouse/cells**

| Category | No. of papers | Compound/ Drug | PXB-mouse | FRG mouse | TK-NOG mouse | uPA/SCID mouse (BE)* | PXB-cells | Publications |
|---|---|---|---|---|---|---|---|---|
| ADME | 48 | Butyrylfentanyl | | | | | O | Kanamori T, et al. Metabolism of Butyrylfentanyl in Fresh Human Hepatocytes: Chemical Synthesis of Authentic Metabolite Standards for Definitive Identification.Biol Pharm Bull. 2019;42(4):623-630. |
| | | Dibutyl Phthalate | | | O | | | Miura T, et al. Steady-State Human Pharmacokinetics of Monobutyl Phthalate Predicted by Physiologically Based Pharmacokinetic Modeling Using Single-Dose Data from Humanized-Liver Mice Orally Administered with Dibutyl Phthalate. Chem Res Toxicol. 2019 Feb 18;32(2):333-340. |
| | | diisononyl phthalate | | | O | | | Iwata H, et al. Predictability of human pharmacokinetics of diisononyl phthalate (DINP) using chimeric mice with humanized liver. Xenobiotica. 2019 Nov;49(11):1311-1322. |
| | | Diclofenac, ketoprofen, nicardipine, pravastatin, salbutamol, warfarin, Acetaminophen, antipyrine, digitoxin, ibuprofen, itraconazole, naproxen, nifedipine, nitrendipine, phenytoin, zolpidem, Amiodarone and fexofenadine | O | | | | | Nakayama K, et al. Prediction of human pharmacokinetics of typical compounds by a physiologically based method using chimeric mice with humanized liver. Xenobiotica. 2019 Apr;49(4):404-414. |
| | | amiodarone, aripiprazole, chlorthalidone, gavestinel, mefloquine, pazopanib, phenobarbital, probucol, pyrimethamine, tamoxifen, UCN-01, warfarin,compound A, compound B. | O | | | | | Miyamoto M, et al. Prediction of human pharmacokinetics of long half-life compounds using chimeric mice with humanised liver. Xenobiotica. 2019 Dec;49(12):1379-1387. |
| | | fenclozic acid | O | | | | | Ekdahl A, et al. The metabolic fate of fenclozic acid in chimeric mice with a humanized liver. Arch Toxicol. 2018 Sep;92(9):2819-2828. |
| | | fentanyl, acetylfentanyl | | | | | O | Kanamori T, et al. Use of hepatocytes isolated from a liver-humanized mouse for studies on the metabolism of drugs: application to the metabolism of fentanyl and acetylfentanyl. Forensic Toxicol. 2018;36(2):467-475. |
| | | Benzydamine | | | O | | | Yamazaki-Nishioka M, et al. Human Plasma Metabolic Profiles of Benzydamine, a Flavin-containing Monooxygenase Probe Substrate, Simulated with Pharmacokinetic Data from Control and Humanized-liver Mice. Xenobiotica. 2018 Feb;48(2):117-123. |
| | | organophosphate or carbamate | | | O | | | Suemizu H, et al. Chimeric Mice with Humanized Liver as a Model for Testing Organophosphate and Carbamate Pesticide Exposure. Pest Manag Sci. 2018 Jun;74(6):1424-1430. |
| | | Diisononyl phthalate | | | O | | | Miura T, et al. Human urinary concentrations of monoisononyl phthalate estimated using physiologically based pharmacokinetic modeling and experimental pharmacokinetics in humanized-liver mice orally administered with diisononyl phthalate. Xenobiotica. 2018 May 18:1-8. |

| | | | | | | |
|---|---|---|---|---|---|---|
| Diclofenac | | O | | | | Wilson CE, et al. The pharmacokinetics and metabolism of diclofenac in chimeric humanized and murinized FRG mice. Arch Toxicol. 2018 Jun;92(6):1953-1967. |
| O$^6$-benzylguanine, and its AOX-derived metabolite, 8-oxo-O$^6$-benzylguanine | O | | | | | Takaoka N, et al. Inhibitory effects of drugs on the metabolic activity of mouse and human aldehyde oxidases and influence on drug-drug interactions. Biochem Pharmacol. 2018 Aug;154:28-38. |
| YM543 | O | | | | | Nakada N. Evaluation of the Utility of Chimeric Mice with Humanized Livers for the Characterization and Profiling of the Metabolites of a Selective Inhibitor (YM543) of the Sodium-Glucose Cotransporter 2. Pharm Res. 2017 Apr;34(4):874-886. |
| pomalidomide | | | O | | | Shimizu M, et al. Metabolic profiles of pomalidomide in human plasma simulated with pharmacokinetic data in control and humanized-liver mice. Xenobiotica. 2017 Oct;47(10):844-848. |
| PF-04937319 | | | O | | | Kamimura H, et al. Simulation of human plasma concentration-time profiles of the partial glucokinase activator PF-04937319 and its disproportionate N-demethylated metabolite using humanized chimeric mice and semi-physiological pharmacokinetic modeling. Xenobiotica. 2017 May;47(5):382-393. . |
| Lumiracoxib | | O | | | | Dickie AP, et al. The pharmacokinetics and metabolism of lumiracoxib in chimeric humanized and murinized FRG mice. Biochem Pharmacol. 2017 Jul 1;135:139-150. |
| Albuterol, Antipyrine, Bazedoxifene, Benzydamine, BIBX1382, Carbazeran, Clonazepam, Dapsone, Diazepam, Diclofenaac, Dolasetron, Entacapone, Fasudil, Gemcitabine, Ibuprofen, Imipramine, Indomethacin, Ketanserin, Ketoprofen, Moxifloxacin, Mycophenolic acid, Naltrexone, O$^6$-Benzylguanine, Pefloxacin, (S)-Naproxen, Sumatriptan, Telmisartan, XK-469, Zaleplon, Zoniporide | O | | | | | Miyamoto M, et al. Comparison of predictability for human pharmacokinetics parameters among monkeys, rats, and chimeric mice with humanised liver. Xenobiotica. 2017 Dec;47(12):1052-1063. |
| Lu AF09535 | O | | | | | Jensen KG, et al. Lack of exposure in a first-in-man study due to aldehyde oxidase metabolism: Investigated by use of 14C-microdose, humanized mice, monkey pharmacokinetics and in vitro methods. Drug Metab Dispos. 2017 Jan;45(1):68-75. |
| Nefazodone | O | | | | | Nakada N, et al. Murine Cyp3a knockout chimeric mice with humanized liver: prediction of the metabolic profile of nefazodone in humans. Biopharm Drug Dispos. 2016 Jan;37(1):3-14. |
| dimethazine | | | | O | | Geldof L, et al. In-vitro and in-vivo metabolism studies of dimethazine. Biomed Chromatogr. 2016 Aug;30(8):1202-1209. |
| prostanozol | | | | O | | Geldof L, et al. Metabolic studies of prostanozol with the uPA-SCID chimeric mouse model and human liver microsomes. Steroids. 2016 Mar;107:139-148. |

| | | | | | | | | |
|---|---|---|---|---|---|---|---|---|
| | | ASP015K | O | | | | | Nakada N, et al. Identification and characterization of metabolites of ASP015K, a novel oral Janus kinase inhibitor, in rats, chimeric mice with humanized liver, and humans. Xenobiotica. 2015;45(9):757-765. |
| | | Thalidomide | | | O | | | Nishiyama S, et al. Simulation of Human Plasma Concentrations of Thalidomide and Primary 5-Hydroxylated Metabolites Explored with Pharmacokinetic Data in Humanized TK-NOG Mice.Chem Res Toxicol. 2015 Nov 16;28(11):2088-2090. |
| | | Diclofenac | | | O | | | Kamimura H,et al. Formation of the Accumulative Human Metabolite and Human-Specific Glutathione Conjugate of Diclofenac in TK-NOG Chimeric Mice with Humanized Livers.Drug Metab Dispos. 2015 Mar;43(3):309-316. |
| | | Diazepam, midazolam, Quinidine, repaglinide, verapamil | O | | | | | Sanoh S, et al. Predictability of plasma concentration-time curves in humans using single-species allometric scaling of chimeric mice with humanized liver. Xenobiotica. 2015;45(7):605-14. |
| | | SUN13834 | O | | | | | Igawa Y, et al. In vitro and in vivo metabolism of a novel chymase inhibitor, SUN13834, and the predictability of human metabolism using mice with humanized liver. Xenobiotica. 2014 Jan;44(2):154-163. |
| | | Diclofenac | O | | | | | Bateman T, et al. Application of Chimeric Mice with Humanized Liver for Study of Human-Specific Drug Metabolism. Drug Metab Dispos. 2014 Jun;42(6):1055-1065. |
| | | Troglitazone | | O | | | | Samuelsson K,  et al. Troglitazone metabolism and transporter effects in chimeric mice: a comparison between chimeric humanized and chimeric murinized FRG mice. Xenobiotica. 2014 Jan;44(2):186-195. |
| | | methylstenbolone | | | | O | | Geldof L, et al. Metabolism of methylstenbolone studied with human liver microsomes and the uPA(+)/(+)-SCID chimeric mouse model. Biomed Chromatogr. 2014 Jul;28(7):974-985. |
| | | zaleplon | O | | | | | Tanoue C, et al. Prediction of human metabolism of the sedative-hypnotic zaleplon using chimeric mice transplanted with human hepatocytes. Xenobiotica. 2013 Nov;43(11):956-962. |
| | | Clemizole | | | O | | | Nishimura T, et al. Using chimeric mice with humanized livers to predict human drug metabolism and a drug-drug interaction. J Pharmacol Exp Ther. 2013 Feb;344(2):388-396. |
| | | FK3453 | O | | | | | Sanoh S, et al. Prediction of human metabolism of FK3453 by aldehyde oxidase using chimeric mice transplanted with human or rat hepatocytes.Drug Metab Dispos. 2012 Dec;40(12):2267-2272. |
| | | Ibuprofen, (S)-naproxen | O | | | | | Sanoh S, et al. Predictability of metabolism of ibuprofen and naproxen using chimeric mice with human hepatocytes. Drug Metab Dispos. 2012 Jan;40(1):76-82. |
| | | Midazolam | O | | | | | Samuelsson K, et al. Pharmacokinetics and metabolism of midazolam in chimeric mice with humanised livers. Xenobiotica. 2012 Nov;42(11):1128-1137. |
| | | Troglitazone | O | | | | | Schulz-Utermoehl T, et al. Evaluation of the pharmacokinetics, biotransformation and hepatic transporter effects of troglitazone in mice with humanized livers. Xenobiotica. 2012 Jun;42(6):503-517. |

| | | | | | | |
|---|---|---|---|---|---|---|
| 6-Deoxypenciclovir, mirtazapine, Dapsone, lamotrigine, salbutamol, sulindac,Diclofenac, Fasudil, (S)-Naproxen, Ibuprofen, ketoprofen, and (S)-warfarin, Zaleplon, ibuprofen, (S)-Naproxen. | O | | | | | Sanoh S, et al. Prediction of in vivo hepatic clearance and half-life of drug candidates in human using chimeric mice with humanized liver. Drug Metab Dispos. 2012 Feb;40(2):322-328. |
| GDC-0834 | O | | | | | Liu L, et al. Significant species difference in amide hydrolysis of GDC-0834, a novel potent and selective Bruton's tyrosine kinase inhibitor.Drug Metab Dispos. 2011 Oct;39(10):1840-9. |
| W695634, SB-406725, GW823093 | O | | | | | De Serres M, et al. Evaluation of a chimeric (uPA+/+)/SCID mouse model with a humanized liver for prediction of human metabolism. Xenobiotica. 2011 Jun;41(6):464-475. |
| promagnon, methylclostebol and methasterone | | | | O | | Lootens L, et al. Metabolic studies with promagnon, methylclostebol and methasterone in the uPA+/+-SCID chimeric mice. J Steroid Biochem Mol Biol. 2011 Nov;127(3-5):374-381. |
| stanozolol | | | | O | | Pozo OJ, et al. Detection and structural investigation of metabolites of stanozolol in human urine by liquid chromatography tandem mass spectrometry. Steroids. 2009 Oct;74(10-11):837-852. |
| methyltestosterone | | | | O | | Pozo OJ, et al. Detection and characterization of a new metabolite of 17alpha-methyltestosterone. Drug Metab Dispos. 2009 Nov;37(11):2153-2162. |
| Methandienone | | | | O | | Pozo OJ, et al. Combination of liquid-chromatography tandem mass spectrometry in different scan modes with human and chimeric mouse urine for the study of steroid metabolism. Drug Test Anal. 2009 Nov;1(11-12):554-567. |
| 4-androstene-3,17-dione | | | | O | | Lootens L, et al. The uPA(+/+)-SCID mouse with humanized liver as a model for in vivo metabolism of 4-androstene-3,17-dione.Drug Metab Dispos. 2009 Dec;37(12):2367-2374. |
| 19-Norandrost-4-ene-3,17-dione | | | | O | | Lootens L, et al. Steroid metabolism in chimeric mice with humanized liver.Drug Test Anal. 2009 Nov;1(11-12):531-537. |
| S-warfarin | O | | | | | Inoue T, et al. CYP2C9-catalyzed metabolism of S-warfarin to 7-hydroxywarfarin in vivo and in vitro in chimeric mice with humanized liver. Drug Metab Dispos. 2008 Dec;36(12):2429-2433. |
| N’-methylnicotinamide | O | | | | | Kitamura S, et al. Aldehyde oxidase-catalyzed metabolism of N1-methylnicotinamide in vivo and in vitro in chimeric mice with humanized liver.Drug Metab Dispos. 2008 Jul;36(7):1202-1205. |
| methandienone | | | | O | | Lootens L, et al. uPA$^{+/+}$-SCID mouse with humanized liver as a model for in vivo metabolism of exogenous steroids: methandienone as a case study. Clin Chem. 2009 Oct;55(10):1783-1793. |
| Cefmetazole | O | | | | | Okumura H, et al. Humanization of excretory pathway in chimeric mice with humanized liver. Toxicol Sci. 2007 Jun;97(2):533-538. |

| | | | | | | | | |
|---|---|---|---|---|---|---|---|---|
| Drug-drug interaction (DDI) | 8 | Rifampicin | O | | | | | Takehara I, et al. Effect of Rifampicin on the Plasma Concentrations of Bile Acid-O-Sulfates in Monkeys and Human Liver-Transplanted Chimeric Mice With or Without Bile Flow Diversion.. J Pharm Sci. 2019 Mar 21. pii: S0022-3549(19)30170-30174. |
| | | VPA and meropenem | | | O | | | Suzuki E, et al. Observation of Clinically Relevant Drug Interaction in Chimeric Mice with Humanized Livers: The Case of Valproic Acid and Carbapenem Antibiotics.Eur J Drug Metab Pharmacokinet. 2017 Dec;42(6):965-972. |
| | | 3-methylcholanthrene, rifampicin | O | | | | | Kakuni M, et al. Chimeric mice with humanized livers: a unique tool for in vivo and in vitro enzyme induction studies.Int J Mol Sci. 2013 Dec 20;15(1):58-74. |
| | | clemizole | | | O | | | Nishimura T, et al. Using chimeric mice with humanized livers to predict human drug metabolism and a drug-drug interaction.J Pharmacol Exp Ther. 2013 Feb;344(2):388-396. |
| | | TCDD | O | | | | | Uno S, et al. CYP1A1 and CYP1A2 expression: comparing 'humanized' mouse lines and wild-type mice; comparing human and mouse hepatoma-derived cell lines.Toxicol Appl Pharmacol. 2009 May 15;237(1):119-126. |
| | | dexamethasone | O | | | | | Emoto C, et al. Non-invasive method to detect induction of CYP3A4 in chimeric mice with a humanized liver. Xenobiotica. 2008 Mar;38(3):239-248. |
| | | beta-naphthoflavone, rifampicin | | | | | O | Nishimura M, et al. Induction of human CYP1A2 and CYP3A4 in primary culture of hepatocytes from chimeric mice with humanized liver.Drug Metab Pharmacokinet. 2005 Apr;20(2):121-126. |
| | | 3-methylcholanthrene, rifampicin | O | | | | | Tateno C, et al. Near completely humanized liver in mice shows human-type metabolic responses to drugs.Am J Pathol. 2004 Sep;165(3):901-912. |
| Liver toxicity | 24 | ENU and aflatoxin B1 | O | | | | | Tateno C, et al. Chimeric mice with human hepatocytes: A new system for genotoxicity studies. Mutat Res. 2019 Mar;839:9-12. |
| | | antibody against TRAIL-receptor 2/death receptor 5 | O | | | | | Nihira K, et al. Chimeric mice with humanized livers demonstrate human-specific hepatotoxicity caused by a therapeutic antibody against TRAIL-receptor 2/death receptor 5. Toxicol Sci. 2019 Jan 1;167(1):190-201. . |
| | | styrene | | | O | | | Miura T, et al. Human plasma and liver concentrations of styrene estimated by combining a simple physiologically based pharmacokinetic model with rodent data. J Toxicol Sci. 2019;44(8):543-548. |
| | | Ketoconazole or Rifampicin | O | | | | | Sanoh S, et al. Changes in Bile Acid Concentrations after Administration of Ketoconazole or Rifampicin to Chimeric Mice with Humanized Liver. Biol Pharm Bull. 2019;42(8):1366-1375. |
| | | fungicide procymidone | O | | | | | Tomigahara Y, et al. Lack of human relevance for procymidone's developmental toxicity attributable to species difference in its kinetics and metabolism. J Pestic Sci. 2018 May 20;43(2):114-123. |
| | | fenofibrate | O | | | | | de la Rosa Rodriguez MA, et al. The whole transcriptome effects of the PPARalpha agonist fenofibrate on livers of hepatocyte humanized mice. BMC Genomics. 2018 Jun 7;19(1):443. |
| | | trimethylamine | | | O | | | Shimizu M,et al. Human plasma concentrations of trimethylamine N-oxide extrapolated using pharmacokinetic modeling based on metabolic profiles of deuterium-labeled trimethylamine in humanized-liver mice. J Toxicol Sci. 2018;43(6):387-393. |

| | | | | | | | | |
|---|---|---|---|---|---|---|---|---|
| | | lenalidomide | | | O | | | Murayama N,et al. Association of pharmacokinetic profiles of lenalidomide in human plasma simulated using pharmacokinetic data in humanized-liver mice with liver toxicity detected by human serum albumin RNA. J Toxicol Sci. 2018;43(6):369-375. |
| | | miodarone | O | | | | | Sanoh S, et al. Assessment of amiodarone-induced phospholipidosis in chimeric mice with a humanized liver. J Toxicol Sci. 2017;42(5):589-596. |
| | | momfluorothrin, metofluthrin, Z-CMCA, NaPB | O | | | | | Okuda Y, et al. Evaluation of the human relevance of the constitutive androstane receptor-mediated mode of action for rat hepatocellular tumor formation by the synthetic pyrethroid momfluorothrin. J Toxicol Sci. 2017;42(6):773-788. |
| | | phenobarbital | O | | | | | Ohara A, et al. Candidate genes responsible for early key events of phenobarbital-promoted mouse hepatocellular tumorigenesis based on differentiation of regulating genes between wild type mice and humanized chimeric mice. Toxicol Res (Camb). 2017 Aug 24;6(6):795-813. |
| | | 1,2-dichloropropane | O | | | | | Toyoda Y, et al. Halogenated hydrocarbon solvent-related cholangiocarcinoma risk: biliary excretion of glutathione conjugates of 1,2-dichloropropane evidenced by untargeted metabolomics analysis. Sci Rep. 2016 Apr 18;6:24586. |
| | | troglitazone and flutamide | O | | | | | Yamazaki H, et al. Zone analysis by two-dimensional electrophoresis with accelerator mass spectrometry of in vivo protein bindings of idiosyncratic hepatotoxicants troglitazone and flutamide bioactivated in chimeric mice with humanized liver. Toxicol. Res., 2015, 4:106–111 |
| | | Fenofibrate | O | | | | | Tateno C, et al. Chimeric Mice with Hepatocyte-humanized Liver as an Appropriate Model to Study Human Peroxisome Proliferator-activated Receptor-alpha. Toxicol Pathol. 2015 Feb;43(2):233-248. |
| | | bosentan | | | O | | | Xu D, et al. Chimeric TK-NOG Mice: A Predictive Model for Cholestatic Human Liver Toxicity.J Pharmacol Exp Ther. 2015 Feb;352(2):274-280. |
| | | furosemide | | | O | | | Xu D, et al. Humanized TK-NOG mice can be used to identify drugs that cause animal-specific hepatotoxicity: a case study with furosemide. J Pharmacol Exp Ther. 2015 Jul;354(1):73-78. |
| | | Phenobarbital | O | | | | | Yamada T, et al. Human Hepatocytes Support the Hypertrophic but not the Hyperplastic Response to the Murine Nongenotoxic Hepatocarcinogen Sodium Phenobarbital in an In Vivo Study Using a Chimeric Mouse with Humanized Liver. Toxicol Sci. 2014 Nov;142(1):137-157. |
| | | troglitazone | O | | | | | Barnes AJ, et al. Endogenous and xenobiotic metabolite profiling of liver extracts from SCID and chimeric humanized mice following repeated oral administration of troglitazone. Xenobiotica. 2014 Jan;44(2):174-185. |
| | | troglitazone | O | | | | | Kakuni M, et al. Chimeric mice with a humanized liver as an animal model of troglitazone-induced liver injury. Toxicol Lett. 2012 Oct 2;214(1):9-18. |
| | | troglitazone | O | | | | | Foster JR, et al. Differential effect of troglitazone on the human bile acid transporters, MRP2 and BSEP, in the PXB hepatic chimeric mouse.Toxicol Pathol. 2012 Dec;40(8):1106-1116. |
| | | 5-n-butyl-pyrazolo[1,5-a]pyrimidine | O | | | | | Yamazaki H, et al. Approach for in vivo protein binding of 5-n-butyl-pyrazolo[1,5-a]pyrimidine bioactivated in chimeric mice with humanized liver by two-dimensional electrophoresis with accelerator mass spectrometry.Chem Res Toxicol. 2010 Jan;23(1):152-158. |
| | | acetaminophen | O | | | | | Sato Y, et al. Human hepatocytes can repopulate mouse liver: histopathology of the liver in human hepatocyte-transplanted chimeric mice and toxicologic responses to acetaminophen.Toxicol Pathol. 2008 Jun;36(4):581-591. |

| | | | | | | | | |
|---|---|---|---|---|---|---|---|---|
| | | acetaminophen | O | | | | | Yamamoto T, et al. Evaluation of human hepatocyte chimeric mice as a model for toxicological investigation using panomic approaches--effect of acetaminophen on the expression profiles of proteins and endogenous metabolites in liver, plasma and urine. J Toxicol Sci. 2007 Aug;32(3):205-215. |
| | | bezafibrate | O | | | | | Shoda J, et al. Bezafibrate induces multidrug-resistance P-Glycoprotein 3 expression in cultured human hepatocytes and humanized livers of chimeric mice. Hepatol Res. 2007 Jul;37(7):548-556. |

[*], Ghent University
